# The clinical implications of circulating microRNAs as potential biomarkers in screening oral squamous cell carcinoma

**DOI:** 10.3389/fonc.2022.965357

**Published:** 2022-11-17

**Authors:** Huan Gao, Yi Shen, Zhengyang Feng, Yuxing Cai, Jianxin Yang, Yaqun Zhu, Qiliang Peng

**Affiliations:** ^1^ Center of Stomatology, The Second Affiliated Hospital of Soochow University, Suzhou, China; ^2^ Department of Radiation Oncology, Suzhou Science & Technology Town Hospital, Suzhou, China; ^3^ Department of Oncology, The Second Affiliated Hospital of Soochow University, Suzhou, China; ^4^ Department of Radiotherapy & Oncology, The Second Affiliated Hospital of Soochow University, Suzhou, China; ^5^ Institute of Radiotherapy & Oncology, Soochow University, Suzhou, China

**Keywords:** oral squamous cell carcinoma, microRNAs, biomarkers, diagnosis, circulating

## Abstract

**Background:**

Recent studies have highlighted the biomarker role of circulating miRNAs in oral squamous cell carcinoma (OSCC), indicating their potential application as early diagnostic markers for OSCC. However, the diagnostic results have proven inconclusive. This study was conducted to evaluate the diagnostic value of circulating miRNAs for OSCC diagnosis.

**Methods:**

Eligible published studies were identified by a literature search carried out in several databases by using combinations of keywords associated with OSCC, circulating miRNAs, and diagnosis. The bivariate meta-analysis model was adopted to summarize the pooled parameters. Afterwards, we thoroughly explored the sources of heterogeneity after evaluating the risk of bias.

**Results:**

A total of 60 studies focusing on 41 circulating miRNAs were included. The pooled sensitivity, specificity, and AUC were 0.75 (95%CI: 0.69-0.80), 0.76 (0.70-0.81), 0.82 (0.79-0.85), respectively. Subgroup analyses showed that miRNA combinations were more accurate than single miRNAs. Additionally, plasma may be a better matrix for miRNAs assays in OSCC diagnosis as the plasma-based miRNA assay had a higher level of diagnostic accuracy than serum-based miRNA assay. Subgroup analyses also suggested that using circulating miRNAs for OSCC diagnosis is more effective in Caucasians than in Asian ethnic groups. Finally, circulating miRNA assays based on large sample sizes have superior diagnostic accuracy than small sample sizes.

**Conclusion:**

Circulating miRNAs might be applied as effective surrogate biomarkers for early diagnosis of OSCC. Nevertheless, future larger-scale prospective studies should be performed to enhance the diagnostic efficiency and investigate the miRNA combinations with more pronounced accuracy.

## Introduction

Oral squamous cell carcinoma (OSCC), accounting for more than 90% of total oral cancer, is often diagnosed at advanced stages and is characterized by a low survival rate (1). Approximately, 50-70% of OSCC patients died within 5 years after they were diagnosed with OSCC due to frequent metastases to regional lymph nodes (2). Although surgical and medical treatments for OSCC have been rapidly developed, the therapeutic efficacy and the five-year survival rate remain unsatisfactory. Early detection of OSCC is vitally significant to enhance the survival outcome, as rates as high as 80-90% can be achieved in the early stages of OSCC patients (3). Currently, diagnosis of OSCC still remains a challenge as the procedures mainly depend on imaging and histological biopsy, which are invasive and uncomfortable (4). Although noninvasive screening tools have been employed, no alternative predictors have been proved effective (5). To this regard, there is a need to explore novel and effective biomarkers for more advancement to promote early detection of OSCC and improve the treatment options.

Over the last decades, microRNAs (miRNAs) have emerged as a new set of biomarkers which play a vital part in oral carcinogenesis (6). MiRNAs are a class of endogenous, non-coding, 18-25 nucleotide length single-strand RNAs that regulate the gene expression at the post-transcriptional level and are highly vital for cell growth and proliferation, mediating critical pathways involved in cancer initiation and progression (7). A plethora of reports have shown that abnormal expressions of specific miRNAs are associated with numerus human diseases, and miRNAs may function as oncogenes or tumor suppressors in various cancer types (8). So far, a significant number of studies have been performed to explore the biomarker role of miRNAs in different cancer types and evidence gathered has revealed the potential use of miRNAs as biomarkers in cancer detection and prognosis (9). Moreover, miRNAs are highly stable in rough conditions and their isolation and quantification have been found to be easy, convenient, and reproducible (10). Therefore, these unique properties make miRNAs as promising diagnostic and prognostic biomarkers in malignant tumors including OSCC which can start from non-invasive specimen collection avoiding postoperative pain.

As we all know, the initiation of OSCC may originate from a multistep accumulation of heterogeneous genetic alterations (11, 12). In the past decades, the expression patterns of miRNAs in OSCC have been recognized as new directions in the exploration of oral carcinogenesis (13). Recent reports have also shown a good association between specific miRNA expression and clinical parameters including tumor metastasis, relapse, and survival in OSCC patients (14, 15). Moreover, a number of studies have demonstrated that miRNAs play an important part during the initiation and progression of OSCC (16, 17). Prominently, miR-21 stands out to be frequently associated with OSCC and may serve as a potential biomarker for diagnosis, prognosis or therapeutic target. Therefore, circulating miRNAs may serve as useful biomarkers for early detection of OSCC (18). However, the diagnostic accuracy of miRNA profiles for OSCC was still inconsistent or even contradictory across different studies, which may be caused by different study designs, different sample sources, different sample sizes and different races.

In this study, we performed a comprehensive meta-analysis of previously published miRNA expression profiling studies to evaluate and summarize the clinical results in the literatures, regarding the potential application of circulating miRNAs in plasma or serum as biomarkers for OSCC diagnosis.

## Materials and method

### Guidelines and searches

This study was performed according to the Preferred Reporting Items for Systematic Reviews and Meta-analysis (PRISMA) guidelines. We have developed a comprehensive search strategy to retrieve all available studies from PubMed, Embase, and Web of Science by using different combinations of keywords including oral, tumor, carcinoma, cancer, squamous cell carcinoma, OSCC, microRNAs, miRNAs, miR-, diagnosis, diagnostic, detect, detection, screen, screening, sensitivity, specificity, and ROC curve. The database was searched from its inception up to April 25, 2022. In addition, the reference lists of relevant reviews were independently scanned to avoid missing any potential studies.

### Eligibility criteria

To be eligible for enrollment in the analysis, the studies had to comply with the following inclusion criteria: (1) original studies focused on the diagnostic value of circulating miRNAs in OSCC; (2) the OSCC diagnosis was achieved based on histopathology as the reference test; (3) studies detected the expression of miRNAs in whole blood, plasma, or serum; (4) the studies provided sufficient data for further calculation, including parameters including specificity, sensitivity, and area under the receiver operating characteristic (ROC) curve. Studies were excluded according to these exclusion criteria as follows: (1) studies were published as review articles, meta-analysis, letters, commentaries, or abstracts; (2) studies failed to provide sufficient information to allow construction of two-by-two table; (3) studies were performed on species other than humans; (4) the language was non-English.

### Data extraction and quality assessment

Two investigators (H.G. and Y.S.) independently reviewed the full texts of included studies and gathered baseline characteristic and clinical diagnostic data including (1) study characteristics: first author, year of study, and country; (2) patients’ demographic characteristics: ethnicity, number, mean age, and TNM stage; (3) miRNA features: miRNA signatures, detection methods, and sample types; (4) data used for further analyses: sensitivity and specificity or true positives (TP), true negatives (TN), false positives (FP), false negatives (FN), and area under the curve (AUC) values. In the present study, the qualities of studies were assessed independently by two reviewers using the revised Quality Assessment of Diagnostic Accuracy Studies (QUADAS-2) criteria (19). If disagreements between the two reviewers occur, they will discuss together to achieve a consensus or consult with the third reviewer (Q.P.).

### Statistical analysis

The bivariate meta-analysis model was adopted to summarize the sensitivity, specificity, positive likelihood ratio (PLR), negative likelihood ratio (NLR), and diagnostic odds ratio (DOR), respectively, together with their 95% confidence intervals (95% CIs) (20). Forest plots of summary statistics were constructed using the data from the enrolled studies. Afterwards, the Summary ROC (SROC) curves, which summarize the sensitivity and specificity of each enrolled study for the assessment of the overall diagnostic performance, were also established, while the AUC values were achieved from the constructed SROC curves (21). The Cochrane’s Q test and I^2^ statistic were applied to judge the presence of statistical heterogeneity across studies (22). P-value less than 0.05 and I^2^ value more than 50% indicated an obvious heterogeneity existing in the current study. When statistical heterogeneity existed between studies, subgroup analysis, meta-regression analysis and sensitivity analysis were adopted to seek the possible sources of heterogeneity (23). Moreover, the risk of publication bias of all the included diagnostic accuracy studies was measured by the Deeks’ funnel-plot asymmetry test (24). In our study, statistical analysis was undertaken using Stata 14.0 software. A p-value of <0.05 was considered statistically significant.

## Results

### Search results and characteristics of studies

**Figure 1:**
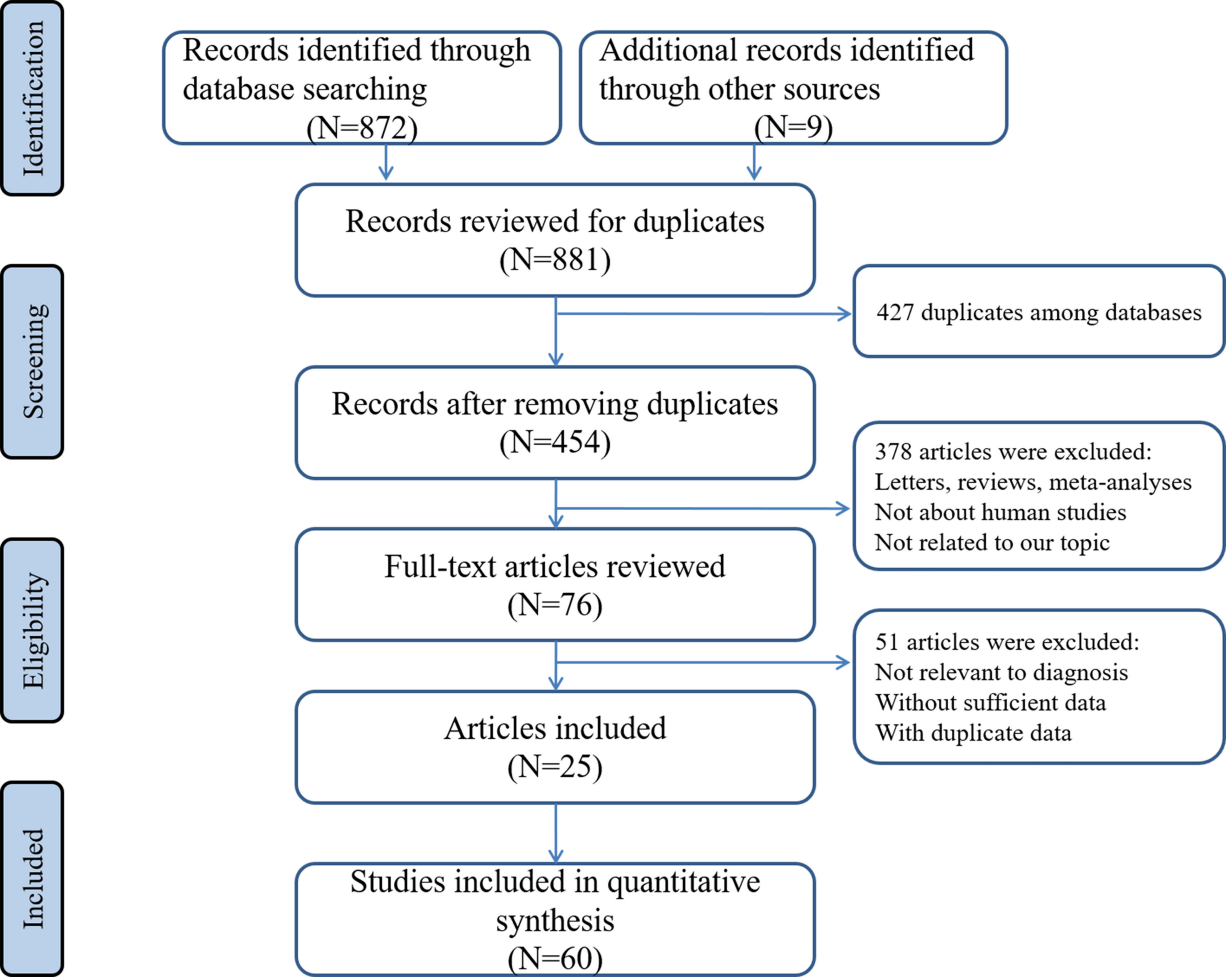
Flow diagram of the identification and selection of the studies.

Through searching the electronic databases with different combinations of the above used keywords, avoiding obviously unrelated titles and abstracts, and after careful consideration of all collected studies, 25 articles involving 60 different miRNAs tests met all inclusion criteria and were included in the final analysis (25–49). A flowchart detailing the selection process was illustrated in **Figure 1**.

All the included studies were performed between 2010 and 2021. Thirty of the selected studies were from China, sixteen from Japan, five from Canada, three from Germany, three from Italy and three from Iran. The number of samples evaluated by each study ranged from 20 to 380. Concerning the type of biological specimen, 32 studies collected from serum, 24 studies from plasma, while 4 studies were collected from whole blood. A total of 55 studies focused on single miRNAs while 5 studies focused on miRNA combinations. All included studies used quantitative real-time reverse transcription-PCR (qRT-PCR) technique to detect the expression of miRNAs. According to the QUADAS-2 assessment tool, the overall analytical quality was acceptable. Details of the enrolled studies in the current study were presented at **Table 1**.

**Table 1:** The main features of the included studies for circulating miRNAs in the diagnosis of OSCC.

Author	Year	Country	Ethnicity	N-p	Age-p	Stage	N-_C_	Age-c	N	Source	miRNA	Methods	AUC	SE	SP
Lin et al	2010	China	Asian	33	NA	I-IV	10	NA	43	Plasma	miR-24	qRT-PCR	0.82	0.70	0.92
Liu et al	2010	China	Asian	43	54	I-IV	21	51	64	Plasma	miR-31	qRT-PCR	0.82	0.63	0.90
Yang et al	2011	China	Asian	34	NA	I-IV	12	NA	46	Plasma	miR-181a	qRT-PCR	0.84	0.94	0.67
Yang et al	2011	China	Asian	34	NA	I-IV	12	NA	46	Plasma	miR-181b	qRT-PCR	0.74	0.90	0.58
Yang et al	2011	China	Asian	34	NA	I-IV	12	NA	46	Plasma	miR-181a, miR-181b	qRT-PCR	0.89	0.78	0.86
Lu et al	2012	China	Asian	54	52	I-IV	36	51	90	Plasma	miR-10b	qRT-PCR	0.932	0.94	0.80
MacLellan et al	2012	Canada	Caucasian	30	63	NA	26	62	56	Serum	miR-338	qRT-PCR	0.82	0.80	0.80
MacLellan et al	2012	Canada	Caucasian	30	63	NA	26	62	56	Serum	miR-29a	qRT-PCR	0.82	0.77	0.77
MacLellan et al	2012	Canada	Caucasian	30	63	NA	26	62	56	Serum	miR-223	qRT-PCR	0.81	0.96	0.60
MacLellan et al	2012	Canada	Caucasian	30	63	NA	26	62	56	Serum	miR-16	qRT-PCR	0.84	0.62	0.93
MacLellan et al	2012	Canada	Caucasian	30	63	NA	26	62	56	Serum	let-7b	qRT-PCR	0.82	0.81	0.80
Hung et al	2013	China	Asian	51	NA	I-IV	12	NA	63	Plasma	miR-146a	qRT-PCR	0.86	0.79	0.92
Liu et al	2013	China	Asian	65	NA	I-IV	24	NA	89	Plasma	miR-196a	qRT-PCR	0.75	0.83	0.68
Liu et al	2013	China	Asian	65	NA	I-IV	24	NA	89	Plasma	miR-196b	qRT-PCR	0.59	0.37	0.98
Ren et al	2014	China	Asian	58	61	I-IV	32	61	90	Blood	miR-21	qRT-PCR	0.788	0.62	0.91
Ries et al	2014	Germany	Caucasian	57	64	I-IV	33	60	90	Blood	miR-186	qRT-PCR	0.69	0.60	0.79
Ries et al	2014	Germany	Caucasian	57	64	I-IV	33	60	90	Blood	miR-3651	qRT-PCR	0.824	0.84	0.67
Ries et al	2014	Germany	Caucasian	57	64	I-IV	33	60	90	Blood	miR-494	qRT-PCR	0.715	0.56	0.82
Lu et al	2015	China	Asian	90	54	I-IV	53	47	143	Plasma	miR-196a	qRT-PCR	0.864	0.67	0.96
Lu et al	2015	China	Asian	90	54	I-IV	53	47	143	Plasma	miR-196b	qRT-PCR	0.96	0.98	0.81
Lu et al	2015	China	Asian	90	54	I-IV	53	47	143	Plasma	miR-196a, miR-196b	qRT-PCR	0.963	0.88	0.92
Tachibana et al	2016	Japan	Asian	31	75	I-IV	31	75	62	Plasma	miR-223	qRT-PCR	0.703	0.68	0.61
Xu et al	2016	China	Asian	101	53	I-IV	103	52	204	Serum	miR-483	qRT-PCR	0.85	0.85	0.75
Liu et al	2017	China	Asian	63	54	I-IV	26	54	89	Plasma	miR-187	qRT-PCR	0.73	0.88	0.50
Chang et al	2018	China	Asian	82	54	I-IV	50	53	132	Plasma	miR-150	qRT-PCR	0.702	0.61	0.77
Chang et al	2018	China	Asian	82	54	I-IV	50	53	132	Plasma	miR-423	qRT-PCR	0.677	0.59	0.73
Chang et al	2018	China	Asian	82	54	I-IV	50	53	132	Plasma	miR-222	qRT-PCR	0.52	0.24	0.87
Chang et al	2018	China	Asian	82	54	I-IV	50	53	132	Plasma	miR-150, miR-423	qRT-PCR	0.749	0.71	0.73
Chen et al	2018	China	Asian	121	NA	I-IV	55	NA	176	Serum	miR-99a	qRT-PCR	0.911	0.80	0.84
Sun et al	2018	China	Asian	80	55	I-III	80	54	160	Plasma	miR-200b	qRT-PCR	0.917	0.90	0.89
Lu et al	2019	China	Asian	82	60	I-IV	53	60	135	Serum	miR-99a	qRT-PCR	0.521	0.87	0.25
Lu et al	2019	China	Asian	82	60	I-IV	53	60	135	Serum	miR-31	qRT-PCR	0.661	0.70	0.52
Lu et al	2019	China	Asian	82	60	I-IV	53	60	135	Serum	miR-138	qRT-PCR	0.547	0.68	0.51
Lu et al	2019	China	Asian	82	60	I-IV	53	60	135	Serum	miR-21	qRT-PCR	0.579	0.64	0.46
Lu et al	2019	China	Asian	82	60	I-IV	53	60	135	Serum	miR-375	qRT-PCR	0.514	0.60	0.50
Lu et al	2019	China	Asian	82	60	I-IV	53	60	135	Serum	miR-99a, miR-31, miR-138, miR-21, miR-375	qRT-PCR	0.776	0.77	0.74
Mahmood et al	2019	China	Asian	100	NA	NA	100	NA	200	Plasma	miR-21	qRT-PCR	0.829	0.91	0.54
Crimi et al	2020	Italy	Caucasian	10	NA	I-IV	10	NA	20	Plasma	miR-133a	qRT-PCR	0.86	0.90	0.80
Crimi et al	2020	Italy	Caucasian	10	NA	I-IV	10	NA	20	Plasma	miR-375	qRT-PCR	0.96	0.80	0.90
Karimi et al	2020	Iran	Caucasian	20	47	NA	20	47	40	Serum	miR-21	qRT-PCR	NA	0.95	0.95
Karimi et al	2020	Iran	Caucasian	20	47	NA	20	47	40	Serum	miR-24	qRT-PCR	NA	0.80	0.70
Karimi et al	2020	Iran	Caucasian	20	47	NA	20	47	40	Serum	miR-29a	qRT-PCR	NA	1.00	1.00
Bigagli et al	2021	Italy	Caucasian	30	65	I-IV	14	51	44	Plasma	miR-210	qRT-PCR	0.951	0.93	0.87
He et al	2021	China	Asian	184	56	I-IV	196	56	380	Plasma	miR-130a	qRT-PCR	0.812	0.99	0.46
Nakamura et al	2021	Japan	Asian	40	67	I-IV	40	64	80	Serum	miR-23	qRT-PCR	0.494	0.75	0.17
Nakamura et al	2021	Japan	Asian	40	67	I-IV	40	64	80	Serum	miR-24	qRT-PCR	0.491	0.60	0.35
Nakamura et al	2021	Japan	Asian	40	67	I-IV	40	64	80	Serum	miR-423	qRT-PCR	0.579	0.88	0.38
Nakamura et al	2021	Japan	Asian	40	67	I-IV	40	64	80	Serum	miR-19a	qRT-PCR	0.659	0.63	0.68
Nakamura et al	2021	Japan	Asian	40	67	I-IV	40	64	80	Serum	miR-19b	qRT-PCR	0.571	0.40	0.88
Nakamura et al	2021	Japan	Asian	40	67	I-IV	40	64	80	Serum	miR-20a	qRT-PCR	0.637	0.75	0.52
Nakamura et al	2021	Japan	Asian	40	67	I-IV	40	64	80	Serum	miR-22	qRT-PCR	0.58	0.63	0.17
Nakamura et al	2021	Japan	Asian	40	67	I-IV	40	64	80	Serum	miR-122	qRT-PCR	0.609	0.20	0.96
Nakamura et al	2021	Japan	Asian	40	67	I-IV	40	64	80	Serum	miR-125	qRT-PCR	0.553	0.47	0.38
Nakamura et al	2021	Japan	Asian	40	67	I-IV	40	64	80	Serum	miR-144	qRT-PCR	0.588	0.45	0.80
Nakamura et al	2021	Japan	Asian	40	67	I-IV	40	64	80	Serum	miR-183	qRT-PCR	0.616	0.45	0.90
Nakamura et al	2021	Japan	Asian	40	67	I-IV	40	64	80	Serum	miR-150	qRT-PCR	0.546	0.22	0.90
Nakamura et al	2021	Japan	Asian	40	67	I-IV	40	64	80	Serum	miR-4419a	qRT-PCR	0.546	0.45	0.77
Nakamura et al	2021	Japan	Asian	40	67	I-IV	40	64	80	Serum	miR-5100	qRT-PCR	0.706	0.50	0.90
Nakamura et al	2021	Japan	Asian	40	67	I-IV	40	64	80	Serum	miR-24, miR-20a, miR-122, miR-150, miR-4419a, miR-5100	qRT-PCR	0.844	0.55	0.93
Wang et al	2021	China	Asian	132	57	I-IV	85	NA	217	Serum	miR-206	qRT-PCR	0.846	0.81	0.73

N-p, number of patients; Age-p, age of patients; N-c, number of controls; Age-c, age of controls; N number, AUC area under curve; SE, sensitivity; SP, Specificity; OSCC, oral squamous cell carcinoma.

### Diagnostic accuracy of circulating miRNAs for OSCC

**Figure 2:**
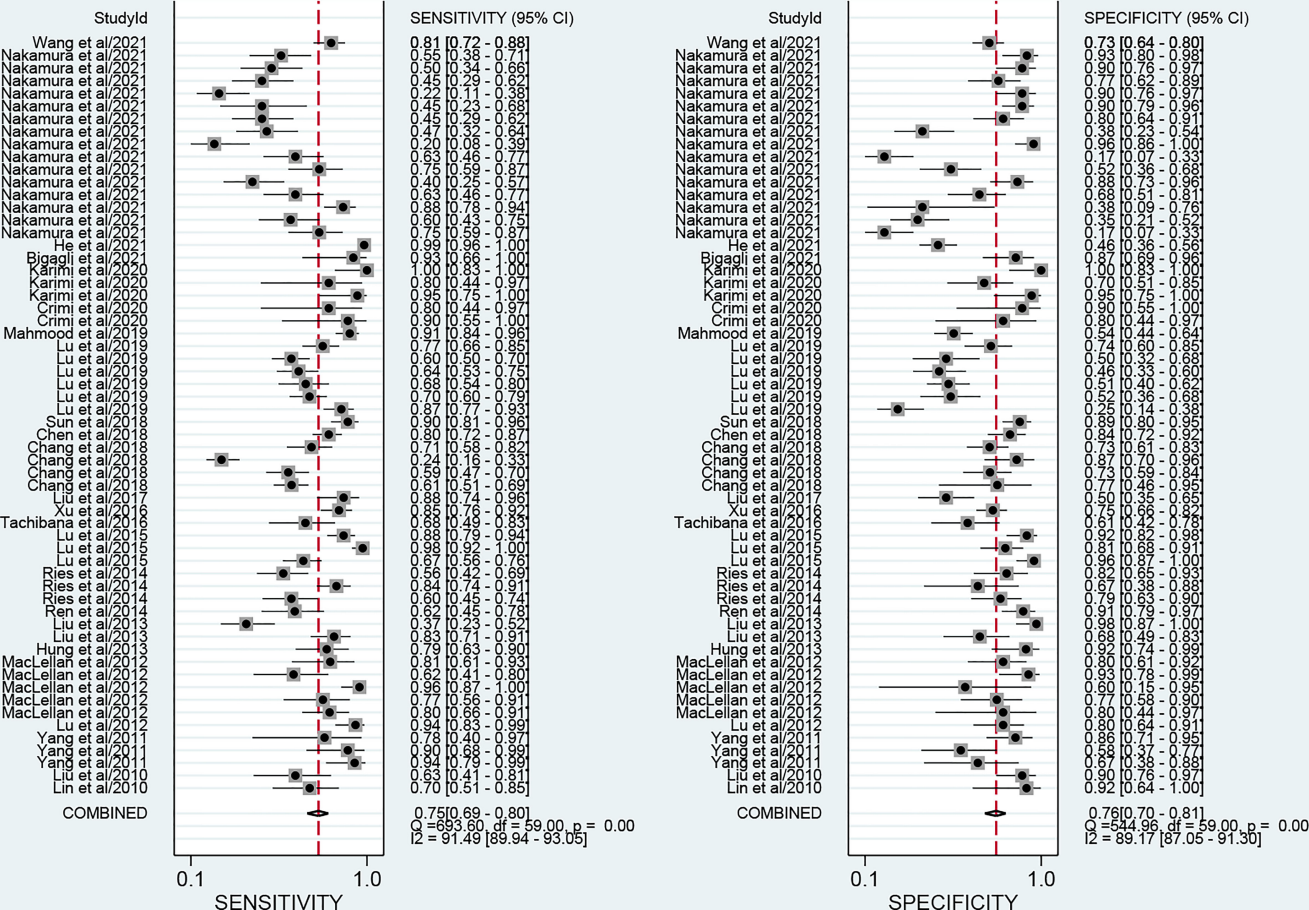
Forest plots of the pooled sensitivity and specificity for circulating miRNAs in detecting OSCC.

**Figure 3:**
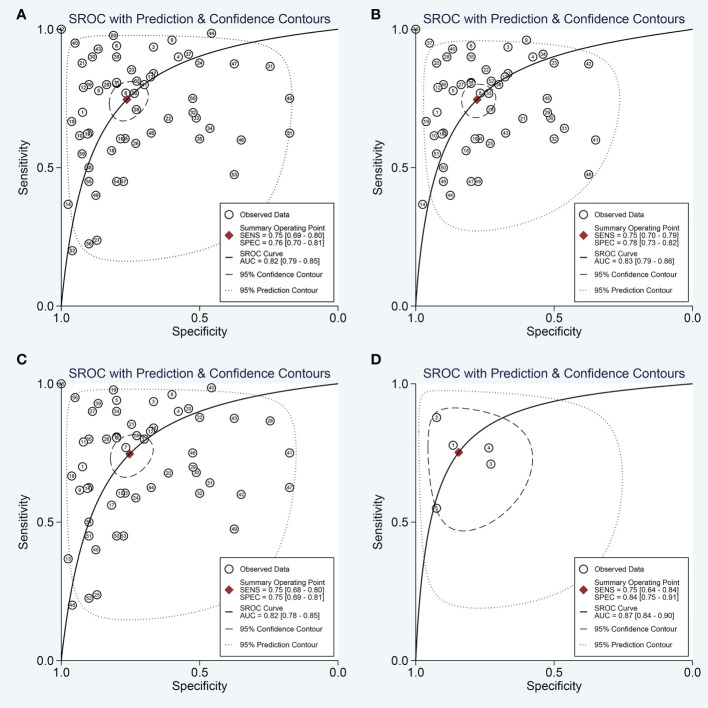
The SROC curve of the pooled analysis. **(A)** SROC curve of overall including the outliers for circulating miRNAs; **(B)** SROC curve of outliers excluded for circulating miRNAs; **(C)** SROC curve for circulating single miRNAs; **(D)** SROC curve for circulating combination miRNAs.

The forest plots describing the pooled sensitivity and specificity for circulating miRNA profiling in distinguishing OSCC patients from normal participants were illustrated at **Figure 2**. Overall, the circulating miRNAs indicated good performances for the detection of OSCC with the pooled sensitivity of 0.75 (95% CI: 0.69-0.80) and the pooled specificity of 0.76 (0.70-0.81). Moreover, the combined PLR, NLR, and DOR were estimated to be 3.2 (2.5-4.0), 0.33 (0.27-0.41), and 9 (6-14), respectively. In addition, the SROC curve of circulating miRNAs in detecting OSCC was presented at **Figure 3A**, and the AUC was calculated to be 0.82 (0.79-0.85).

**Figure 4:**
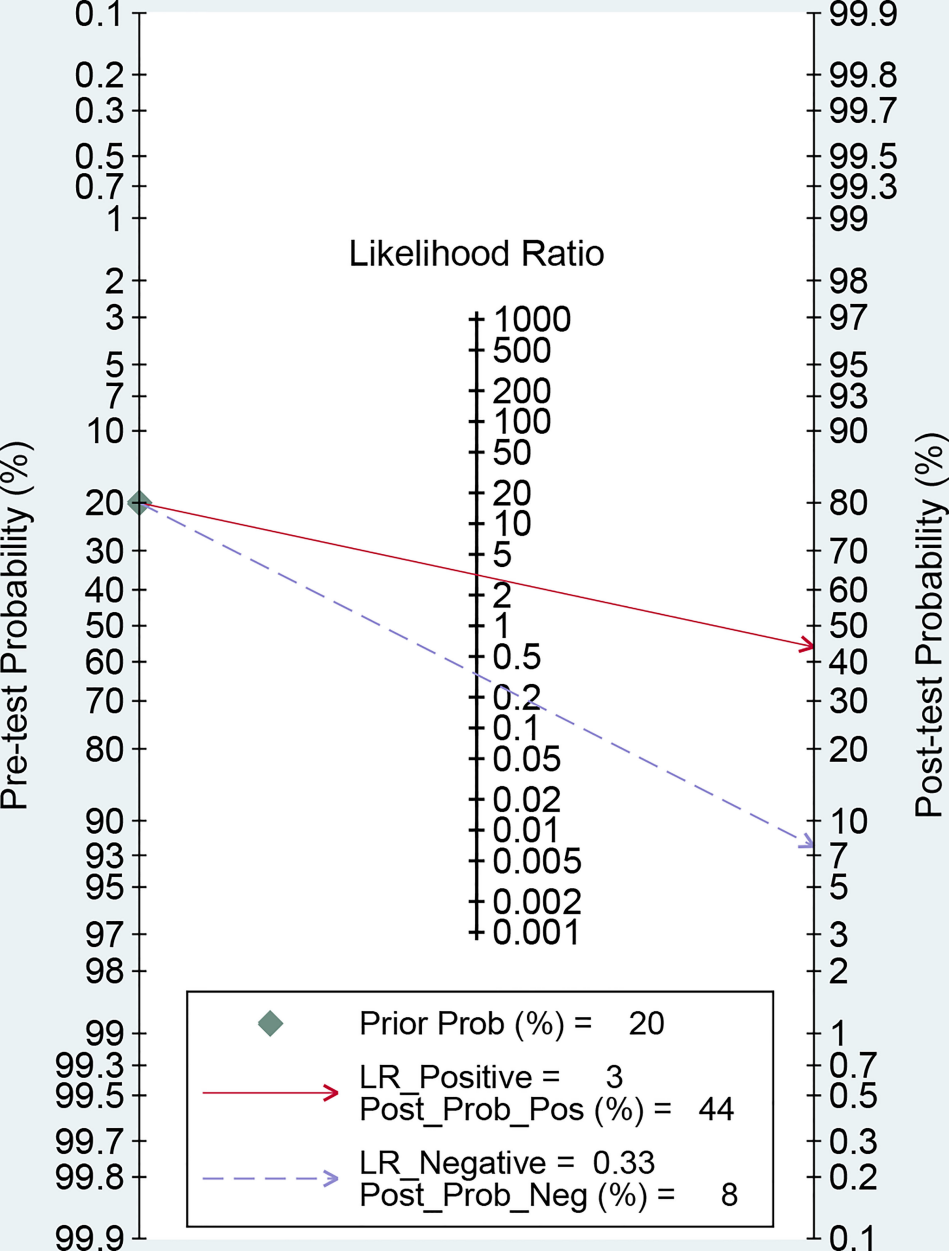
Fagan’s monogram evaluating the clinical utility of circulating miRNAs for differentiating OSCC patients.

Then, the Fagan’s nomogram was utilized to confirm the clinical utility of the circulating miRNAs in OSCC. As illustrated in **Figure 4**, when the pre-test probability of OSCC was 20%, a positive measurement could improve the post-test probability of suffering cancer to 44%, while the post-test probability will reduce to 8% if a negative measurement happened.

The above parameters revealed moderate discriminative ability of the application of circulating miRNAs as biomarkers for the early diagnosis of OSCC.

### Heterogeneity and subgroup analyses

In this study, the heterogeneity analysis using I^2^ suggested significantly higher heterogeneity in both pooled sensitivity (I^2^ = 91.49%; P<0.001) and pooled specificity (I^2^ = 89.17%; P<0.001). We first explored whether the heterogeneity may come from the threshold effect. The Spearman correlation coefficient between the sensitivity logarithm and (1-specificity) logarithm is calculated to be -0.24, and the P-value is 0.06, suggesting no obvious threshold effect.

**Figure 5:**
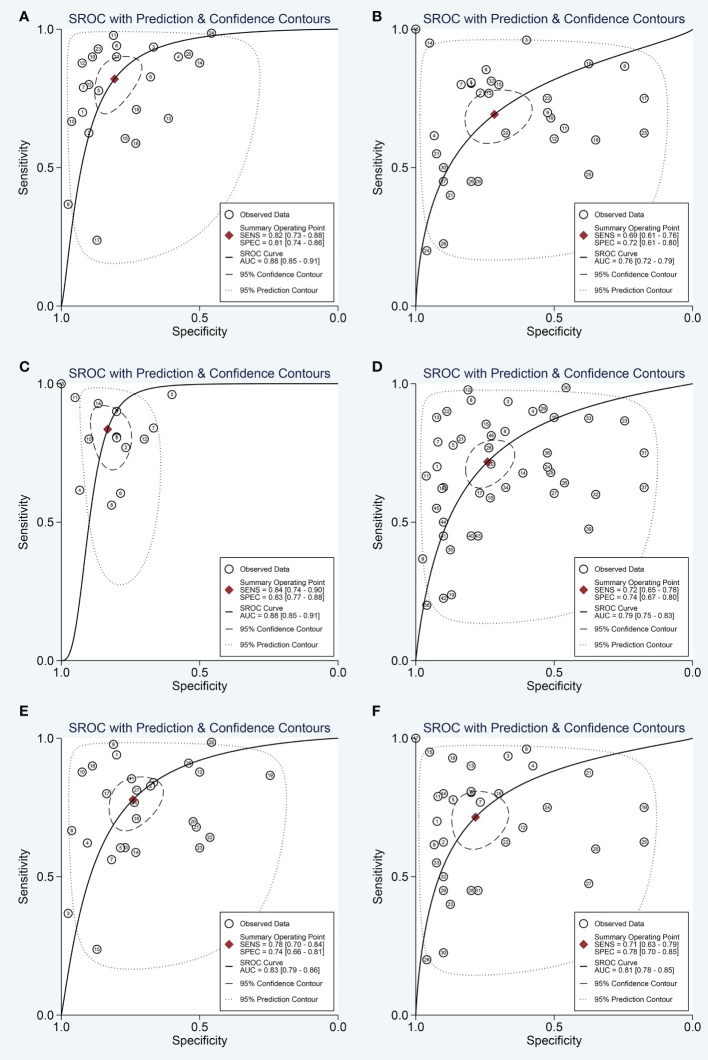
The SROC curve of the pooled analysis by some subgroups. **(A)** SROC curve for plasma miRNAs; **(B)** SROC curve for serum miRNAs; **(C)** SROC curve for circulating miRNAs based on Caucasian population; **(D)** SROC curve for circulating miRNAs based on Asian population; **(E)** SROC curve for circulating miRNAs based on large sample sizes; **(F)** SROC curve for circulating miRNAs based on small sample sizes.

Next, the subgroup analyses were carried out. As exemplified by **Table 2**, miRNA combination testing achieved a more overall promising accuracy than single miRNA assay, with a similar sensitivity of 0.75 (0.64-0.84) versus 0.75 (0.68-0.80), a higher specificity of 0.84 (0.75-0.91) versus 0.75 (0.69-0.81), a higher AUC of 0.87 (0.84-0.90) versus 0.82 (0.78-0.85), respectively. The SROC curves of single miRNAs and combination miRNAs were plotted at **Figures 3C, D**, respectively. Following subgroup analysis depending on sample sources, the plasma-based miRNA assay exhibited a higher level of diagnostic accuracy than serum-based miRNA assay accompanied by a pooled sensitivity of 0.82 (0.73-0.88) versus 0.69 (0.61-0.76), specificity of 0.81 (0.74-0.86) versus 0.72 (0.61-0.80) and AUC of 0.88 (0.85-0.91) versus 0.76 (0.72-0.79). Moreover, the diagnostic accuracy based on ethnicity was also assessed through subgroup analysis. The results indicated that the sensitivity, specificity, and AUC of circulating miRNAs for OSCC detection in the Caucasian group were higher than those in the Asian group due to higher sensitivity: 0.84 (0.74-0.90) versus 0.72 (0.65-0.78), higher specificity: 0.83 (0.77-0.88) versus 0.74 (0.67-0.80), and higher AUC: 0.88 (0.85-0.91) versus 0.79 (0.75-0.83). In addition, we also conducted a subgroup analysis to identify whether sample size had a potential influence on the whole diagnostic value of circulating miRNAs. The stratified analysis by sample size suggested that the diagnostic value of miRNAs based on large sample sizes (N>80) might be more significant than it is for small sample sizes (N ≤ 80), with a pooled sensitivity of 0.78 (0.70-0.84) versus 0.71 (0.63-0.79), specificity of 0.74 (0.66-0.81) versus 0.78 (0.70-0.85) and AUC of 0.83 (0.79-0.86) versus 0.81 (0.78-0.85). The SROC curves of these subgroups were also plotted at **Figure 5**.

**Table 2:** Summary table of the diagnostic accuracy of circulating miRNAs in the diagnosis of OSCC.

Analyses	Studies	Sensitivity (95% CI)	Specificity (95% CI)	DOR (95% CI)	PLR (95% CI)	NLR (95% CI)	AUC (95% CI)
Sample types							
Serum	32	0.69 (0.61-0.76)	0.72 (0.61-0.80)	6 (3-10)	2.4 (1.8-3.4)	0.43 (0.33-0.55)	0.76 (0.72-0.79)
Plasma	24	0.82 (0.73-0.88)	0.81 (0.74-0.86)	19 (12-32)	4.3 (3.2-5.8)	0.22 (0.15-0.33)	0.88 (0.85-0.91)
Whole blood	4	0.67 (0.53-0.78)	0.81 (0.70-0.89)	9 (5-15)	3.6 (2.4-5.4)	0.41 (0.30-0.57)	0.82 (0.78-0.85)
Ethnicity							
Asians	46	0.72 (0.65-0.78)	0.74 (0.67-0.80)	7 (5-11)	2.8 (2.1-3.6)	0.38 (0.30-0.48)	0.79 (0.75-0.83)
Caucasians	14	0.84 (0.74-0.90)	0.83 (0.77-0.88)	25 (12-54)	5.0 (3.5-7.0)	0.20 (0.12-0.33)	0.88 (0.85-0.91)
Sample size							
Large sample size (>80)	27	0.78 (0.70-0.84)	0.74 (0.66-0.81)	10 (6-16)	3.0 (2.3-4.0)	0.30 (0.22-0.41)	0.83 (0.79-0.86)
Small sample size (≤80)	33	0.71 (0.63-0.79)	0.78 (0.70-0.85)	9 (5-16)	3.3 (2.3-4.8)	0.36 (0.27-0.49)	0.81 (0.78-0.85)
miRNA profiling							
Single	55	0.75 (0.68-0.80)	0.75 (0.69-0.81)	9 (6-13)	3.0 (2.4-3.8)	0.34 (0.27-0.43)	0.82 (0.78-0.85)
Combination	5	0.75 (0.64-0.84)	0.84 (0.75-0.91)	17 (7-39)	4.8 (2.8-8.4)	0.29 (0.19-0.45)	0.87 (0.84-0.90)
Overall	60	0.75 (0.78-0.93)	0.76 (0.70-0.81)	9 (6-14)	3.2 (2.5-4.0)	0.33 (0.27-0.41)	0.82 (0.79-0.85)
Outliers excluded	52	0.75 (0.70-0.79)	0.78 (0.73-0.82)	10 (7-15)	3.4 (2.7-4.2)	0.33 (0.27-0.40)	0.83 (0.79-0.86)

DOR, diagnostic odds ratio; PLR, positive likelihood ratio; NLR, negative likelihood ratio; AUC, area under curve; OSCC, oral squamous cell carcinoma.

### Influence analysis and meta-regression

**Figure 6:**
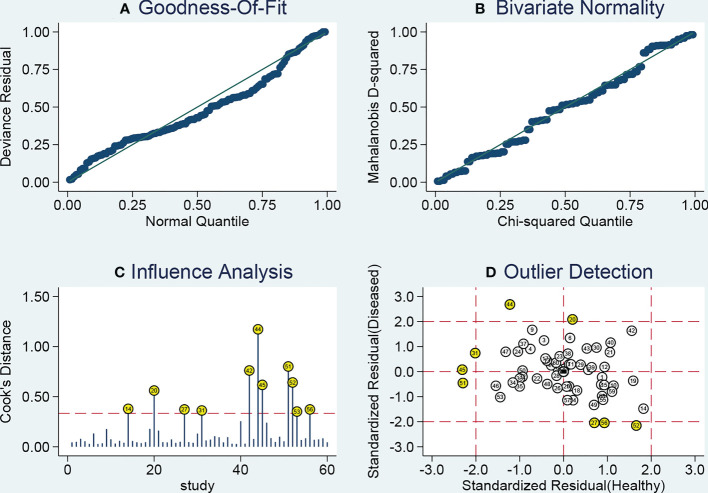
Sensitivity analysis results. **(A)** Goodness of fit; **(B)** bivariate normality; **(C)** influence analysis; **(D)** outlier detection.

As heterogeneity observed in the present study, sensitivity analysis was performed (**Figure 6**). The influence analysis and outlier detection identified several outlier individual studies. After excluding seven outliers (34, 38, 41, 47, 48), the I^2^ for heterogeneity decreased both in sensitivity (from 91.49% to 84.30%) and specificity (from 89.17% to 84.31%). However, the overall results showed only minimal changes and were similar to the original ones as the pooled specificity of the overall study increased from 0.76 to 0.78, PLR increased from 3.2 to 3.4, DOR decreased from 9 to 10, and AUC increased from 0.82 to 0.83, while sensitivity and NLR had no changes. The SROC curve of outliers excluded for circulating miRNAs was plotted at **Figure 3B**. The above results suggested that our study was robust.

Furthermore, a meta-regression analysis was applied to distinguish the potential sources of heterogeneity across studies by exploring research characteristics including specimen type, sample size, and ethnicity. The results suggested that ethnicity may be responsible for the heterogeneity while other factors reveled low likelihood of sources of inter-study heterogeneity.

### Publication bias

**Figure 7:**
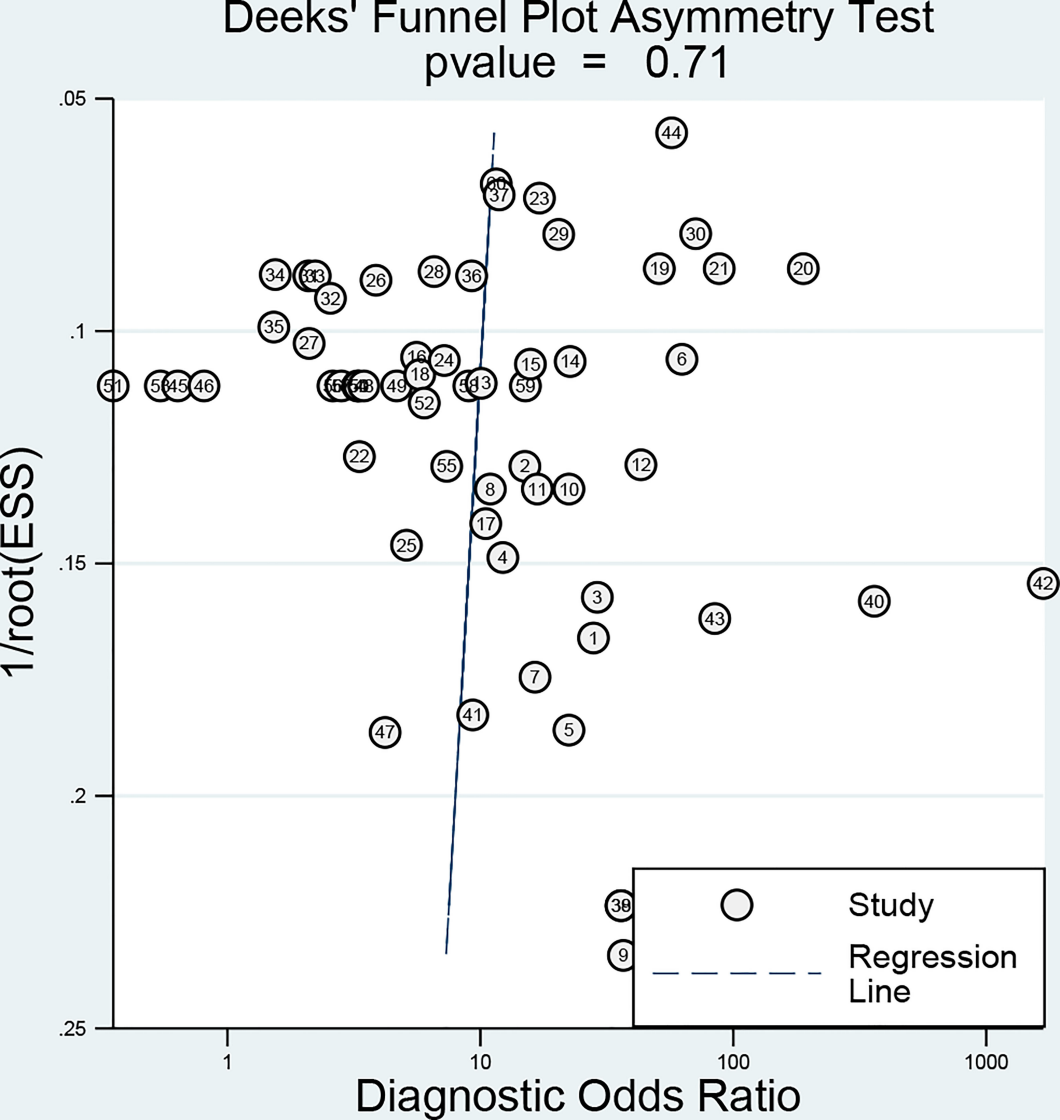
Deeks’ funnel plots for the assessment of potential bias.

Deeks’ funnel-plot asymmetry test (**Figure 7**) was performed to investigate the potential publication bias. The assessment of publication bias indicated that the funnel plots were asymmetric with a slope coefficient P-value calculated to be 0.71 in the overall studies, indicating there was no obvious publication bias in the current study.

## Discussion

Despite great advancement in the treatment modalities, the survival rate of patients with OSCC is still low. Early detection is of great clinical importance for the optimization of treatment strategies and improvement of long-term survival for OSCC patients. Although histopathological investigation is still recognized as the gold standard for clinicians, it is an invasive examination procedure that is temporally and spatially restricted and unable to indicate the whole molecular landscape of OSCC. Thus, it is indispensable to explore less invasive, reliable, and effective detection methods to enhance early OSCC detection. Currently, the miRNA detection in different biofluids has been explored as novel diagnostic biomarkers for OSCC. However, the clinical value of circulating miRNAs for OSCC diagnosis remains unclear. Due to the inconsistencies of previously published studies, we performed this comprehensive study to determine whether circulating miRNAs could be applied as a non-invasive and effective method in the early screening of OSCC.

To our knowledge, this is the most comprehensive study ever conducted on the role of circulating miRNAs in OSCC. A total of sixty studies were identified eligible and enrolled for the assessment of the diagnostic value of circulating miRNAs in OSCC. The overall analysis demonstrates moderate diagnostic accuracy as circulating miRNA profiling harbored a relatively high pooled diagnostic value in detecting OSCC, where the combined sensitivity and specificity were 0.75 and 0.76, respectively, corresponding with an AUC of 0.82. The stratified analysis by sample sources indicated that the plasma-based assay seemed to undergo a higher pooled sensitivity, specificity, and AUC than serum-based assay. Our results provided evidence that plasma may be a better matrix for diagnostic profiling of miRNAs in OSCC, which was also consistent with previous studies (50). Moreover, previous studies have suggested that genetic background may have some effect on the miRNA concentrations in body fluids or tumor lesions and the biomarker value of miRNAs varies among different ethnicities (51). In our pooled results by subgroup analysis, we found that the Caucasian population-based miRNA test harbored an overall higher accuracy compared than that of the Asians, suggesting that biomarker performance for circulating miRNAs in OSCC diagnosis may also be determined by different genetic background. Remarkably, the impact of genetic background on the diagnostic value of circulating miRNAs was also confirmed by a meta-regression analysis. Additionally, our previous study also reported that the diagnostic accuracies of miRNAs in different cancers were affected by sample sizes (52). More importantly, we observed a higher diagnostic accuracy in miRNA detection tests based on large sample size (n>80) than investigations with small sample sizes (n ≤ 80), which may suggest that future translational and clinical investigations concentrating on large-scale prospective studies are worth performing to validate the diagnostic efficacy of circulating miRNAs in OSCC.

Accumulating evidence described that tumorigenesis is a complex cell processes involving multiple miRNAs (53). For OSCC, it is a highly heterogeneous cancer with a complicated etiology and single miRNA is hard to diagnose OSCC with satisfactory performance (11). Combination biomarkers may be more comprehensive than single maker in dictating the complicated cancer evolutionary process and may be more powerful in cancer detection (54–56). There have been promising results yielded from studies that combination miRNAs had a higher level of diagnostic power than single miRNAs. However, since there were only five tests focusing on miRNA combination biomarkers in detecting OSCC, we could not answer the question that which and how many miRNAs should be combined together to enhance the diagnostic accuracy. This indicates that further research should be concentrated on the combined use of miRNA panels to investigate their effect on diagnostic accuracy.

There were 41 miRNAs utilized as diagnostic biomarkers involved in the present study, which may provide new insight into the early detection of OSCC. However, the exact role of miRNAs in the carcinogenesis of OSCC remains unclear. Recently, several researchers have demonstrated a functional role for miRNAs in the initiation and progression of OSCC, which may help us understand the potential biomarker role of miRNAs in OSCC. For example, Lin et al. reported that miR-31 played a pivotal part during the progression of OSCC through establishing a complicated network with its regulated genes and the signaling cascades such as EGF-AKT signaling axis, Hippo pathway, and Wnt signaling (57). Data from the study by Peng et al. revealed that downregulation of miR-130a could inhibit OSCC proliferation and metastasis by the Hippo-YAP pathway, which may provide potential target for OSCC therapy (58). Previous studies have also demonstrated that miR-133a plays tumor suppressive role in OSCC by inhibiting the Notch signaling pathway *via* binding to CTBP2 (59). Moreover, mechanism research has suggested that miR-222 affects OSCC cell proliferation, migration, invasion, and apoptosis by targeting CDKN1B (60). In addition, previous evidence has demonstrated that miR-144 inhibits tumorigenesis of OSCC by targeting ERO1L/STAT3 signaling pathway (61). Besides, available data suggest that the expression of miR-196a increased in OSCC cells against normal oral squamous cells and downregulation of miR-196a could inhibit the malignant biological processes of OSCC cells through targeting FOXO1 (62). In a word, the altered expressions of miRNAs and the involved pathways may contribute to the biological behaviors in OSCC, ultimately leading to tumorigenesis. However, as for more detailed mechanisms of miRNAs in OSCC, further studies are needed to make further investigations.

Since circulating miRNAs possess the distinctive advantages of tumor specificity, stable, extracted easily and non-invasive, they may be applied as perfect non-invasive biomarkers for cancer detection. Our study also demonstrated that circulating miRNAs may represent potentially promising biomarkers for the detection of OSCC. However, there is still a long way to go before they can be used in clinical practice in OSCC diagnosis. For example, different studies from different laboratories or testing platforms used diverse cut-off values and different normalization in the detection of miRNAs, which may potentially affect the diagnostic efficacy and generate heterogeneity and uncertainty. Therefore, a consensus in the scientific community should be indispensable to establish the optimum cut-off values and detection methods. Moreover, current studies on miRNAs detection in OSCC were isolated and the future exploration of miRNA biomarker for OSCC should be rooted in systematical and dynamical manner to develop integrative diagnostic models based on specific miRNAs and combinations with more appropriate and better prediction capacity. In addition, studies included in our studies are still far from enough and the size of available samples in the individual studies are still relatively small. Thus, large-scale and well-designed prospective randomized controlled studies are required to confirm the actual diagnostic value of circulating miRNA assays for OSCC and promote the clinical application. Moreover, we believe that large adequately designed prospective studies may help determine a specific miRNA type or specific miRNA combinations that may be more appropriate and better used in clinical decision-making for OSCC patients.

The present study has some limitations that must be acknowledged. First, we did observe significant heterogeneity because of discrepancies among the different studies. Although several common check methods of evaluating the risk of bias assessment including subgroup, meta-regression, and sensitivity analysis were performed, we were still unable to clarify the precise factors contributing to the significant heterogeneity. Second, since some clinical factors such as gender difference, age distribution, and TNM stage were not detailed in some studies, we could not carry out any other subgroup analysis based on them. Third, though we had planned to investigate the impact of cut-off value on heterogeneity by using the meta-regression analyses, this could not be accomplished due to insufficient data and the different standards in different enrolled diagnostic tests. Moreover, as discussed in the subgroup analyses and meta-regression analysis, ethnicity might be considered as a source of heterogeneity among individual studies. However, all the diagnostic miRNA tests were conducted on the basis of Asian and Caucasian population, whereas African population should have been enrolled. Finally, the history of tobacco and alcohol consumption may influence the study results. However, these data have not been provided by some of the included studies and we cannot perform such analysis. Regardless of these limitations, ours is the most comprehensive study that aggregated available data on circulating miRNAs and analyzed their application in the field of OSCC detection.

## Conclusions

In conclusion, our results indicated that circulating miRNAs has a relatively high diagnostic accuracy in the detection of OSCC and might be applied in noninvasive screening tests for OSCC. In addition, combination miRNA biomarkers have exhibited a prominent advantage over single miRNAs in improving diagnostic accuracy of OSCC. However, further translational and clinical investigations based on large-scale prospective studies are necessary to validate the diagnostic efficacy of circulating miRNAs and promote their clinical application in OSCC.

## Data availability statement

The original contributions presented in the study are included in the article. Further inquiries can be directed to the corresponding author.

## Author contributions

HG, YS and ZF performed the computational analysis, and wrote the manuscript. YC, JY and YZ were responsible for the statistical analysis in meta-analysis part. QP conceived of the study, and took part in its design and coordination. All authors contributed to the article and approved the submitted version.

## Funding

This work was supported by Suzhou Radiotherapy Clinical Medical Center (Szlcyxzx202103), Scientific Research Program for Young Talents of China National Nuclear Corporation (QP) and Leader Project of Clinical Technology Application Research for Jiangsu Gerontology (LR2021023).

## Acknowledgments

We would like to thank the reviewers and editors for their constructive comments.

## Conflict of interest

The authors declare that the research was conducted in the absence of any commercial or financial relationships that could be construed as a potential conflict of interest.

## Publisher’s note

All claims expressed in this article are solely those of the authors and do not necessarily represent those of their affiliated organizations, or those of the publisher, the editors and the reviewers. Any product that may be evaluated in this article, or claim that may be made by its manufacturer, is not guaranteed or endorsed by the publisher.
